# Graph Representation Integrating Signals for Emotion Recognition and Analysis

**DOI:** 10.3390/s21124035

**Published:** 2021-06-11

**Authors:** Teresa Zawadzka, Tomasz Wierciński, Grzegorz Meller, Mateusz Rock, Robert Zwierzycki, Michał R. Wróbel

**Affiliations:** Faculty of Electronics, Telecommunications and Informatics, Gdańsk University of Technology, 80-233 Gdańsk, Poland; tomaszwiercinski26@gmail.com (T.W.); grzegorz.meller@outlook.com (G.M.); mateuszrockm@gmail.com (M.R.); robert.zwierzycki@gmail.com (R.Z.)

**Keywords:** affective computing, biosignals, datasets, emotion recognition, graph databases, signal integration

## Abstract

Data reusability is an important feature of current research, just in every field of science. Modern research in Affective Computing, often rely on datasets containing experiments-originated data such as biosignals, video clips, or images. Moreover, conducting experiments with a vast number of participants to build datasets for Affective Computing research is time-consuming and expensive. Therefore, it is extremely important to provide solutions allowing one to (re)use data from a variety of sources, which usually demands data integration. This paper presents the Graph Representation Integrating Signals for Emotion Recognition and Analysis (GRISERA) framework, which provides a persistent model for storing integrated signals and methods for its creation. To the best of our knowledge, this is the first approach in Affective Computing field that addresses the problem of integrating data from multiple experiments, storing it in a consistent way, and providing query patterns for data retrieval. The proposed framework is based on the standardized graph model, which is known to be highly suitable for signal processing purposes. The validation proved that data from the well-known AMIGOS dataset can be stored in the GRISERA framework and later retrieved for training deep learning models. Furthermore, the second case study proved that it is possible to integrate signals from multiple sources (AMIGOS, ASCERTAIN, and DEAP) into GRISERA and retrieve them for further statistical analysis.

## 1. Introduction

With the increase of research in Affective Computing field [1], the number of published datasets related to emotion processing from experiments is growing [2]. They include biosignals like EEG, ECG, GSR or facial expressions, along with contextual information and sometimes emotional states. Such data can be acquired in different ways e.g., from multiple devices with various sensors or labelling by different annotators, and in the case of emotional states also from external recognition methods (all this data, if it is not misleading are further called experiment originating data).

However, the data are dispersed, stored in plenty of formats, different datasets provide diverse biosignals and emotional states which are retrieved with various frequencies or even irregularly spaced. Such circumstances, connected with a lack of unified storage for experiment originating data and semi-automatic methods of integrating them, make it difficult to use data from various sources to conduct new research in the field of Affective Computing.

To address this problem, we propose a GRISERA (Graph Representation Integrating Signals for Emotion Recognition and Analysis) framework for integrating experiment originated data based on a standardized graph model, which is an original contribution of this work. The paper presents and discusses the model and the methods of creating it. As it has already been shown in [3] the graph representation very well suits the purposes of signal processing and thus it is a natural candidate for the integration of data with the presented characteristics [4].

The main objective of our research is to develop a comprehensive framework that integrates data from various emotion recognition experiments, stores them in a graph database, and makes them available for emotion analysis and recognition.

For this purpose we have defined 2 Research Questions:

- RQ1: Does the use of graph data representation make it possible to retrieve data from different experiments in a unified way?
- RQ2: Does the graph representation of the relationship between biosignals, identified emotional states, and contextual information allow for the extraction of useful data for Affective Computing research?

Research question RQ1 addresses the problem of integrating biosignals, contextual information and emotional estimates from different experiments, regardless of the devices and sensors used. The ability to extract data in a unified manner from multiple sources, independently of their characteristics, will facilitate analysis in the field of emotion recognition. Moreover, the flexibility aspect is addressed i.e., in what way the proposed representation can be expanded for new types of biosignals, not previously identified.

Regarding the RQ2, the question covers the problem of using the data collected in the graph database for the analysis and recognition of emotions. In this context, the relationships between biosignals recorded by sensors and also between biosignals and other data, such as emotion states or contextual information, is particularly relevant.

The GRISERA framework is presented in this paper. In the beginning, the detailed motivations of our work are presented (Section 2), followed by the description of the methodology used in the rest of the paper (Section 3). Later, Section 4 discusses other research concerning the problem of signal integration in the field of Affective Computing. Then, in Section 5 the graph representation is described with relation to various biosignals and emotional state models as well as contextual information, in Section 6 process of GRISERA creation is described and in Section 7 data retrival approach is presented. In Section 8 validation case studies are presented, followed by a discussion of GRISERA framework validity in Section 9 and outline of challenges that might be addressed by future works in Section 10.

## 2. Motivations

For the purpose of motivating the research problem for which we believe the GRISERA framework is the answer, we use the Research Data Universe classification proposed by Thanos [5], which was based on the work of the National Research Council and National Science Board [6,7,8]. Comparing the proposed guidelines with the state of research on Affective Computing, it can be deduced that the greatest immaturity lies in the area of databases and datasets. The National Science Board has grouped the data collections into three categories [8]:

- Research Data Collections—data from one or more research projects, typically containing data that can only be processed to a limited extent,
- Resource of Community Data Collections—collections of a single scientific community, often setting internal standards.
- Reference Data Collections—datasets used by numerous scientific community groups, introducing well-established, comprehensive standards.

The datasets in Affective Computing field mainly belong to Resource of Community Data Collections, but the state of this category is still immature. Even datasets serve the community of AC researchers and there are some standards like emotional states models, there are no reusable lexicons and ontologies [9] as well as unified data representations [10].

Therefore, a number of decisions need to be made before building data collections in Affective Computing research to take it to the next level of maturity:

- **$D_{1}$**—the data are to be obtained through planned and performed experiments dedicated to the conducted research?
- **$D_{2}$**—the data obtained from experiments are planned to be published so they can be used by other researchers?
- **$D_{3}$**—the data are planned to be used only for the current study or it is planned to reuse them in other ones?
- **$D_{4}$**—the data are to be obtained from one or more experiments?
- **$D_{5}$**—the data obtained from external resources come from one or more data sets?

The first decision ($D_{1}$) is mainly connected with the type of research data. It results in preparation of experimental data that can be raw data consisting of original observations, derivative data generated by processing activities or verified data generated by curatorial activities [5]. In the field of Affective Computing experiment data are mainly raw (e.g., raw ECG or GSR signal) and derivative (e.g., HRV for ECG or number of peaks for GSR signal), often enriched with computational data produced by executing a computer model or simulation (e.g., recognized emotional states). The experiment data in AC has also some features of observational data, as often they base on participant observation, what is difficult to recollect, and in some situations even impossible (the reaction of the specified person can vary between the first and the second observation even in the same recreated conditions).

The second decision ($D_{2}$) is strongly connected with the functional category of data collections to be prepared and their actors [8]. In this regard, it is necessary to develop community-wide standards so that the data can be easily used by other Affective Computing researchers.

The next three decisions ($D_{3}$, $D_{4}$ and $D_{5}$) correspond to data uses aspect of research-data universe. They are defined in [5] as end use (the ability to access a dataset to verify some fact or perform some other operations) and derivative use (the capability to produce a new dataset from an existing one, which can be used for the same, similar or a completely different purpose).

The GRISERA framework is intended to be the first step and for our best knowledge first approach in the field Affective Computing to address the presented issues by:

- introducing a new representation for experiment-originated data in AC,
- introducing the solution that allows representing raw and derivative data as well as computational data in a consistent way,
- providing the query patterns for data retrieving,
- addressing base aspects of data integration.

## 3. Methodology

The GRISERA framework bases on the graph representation of experiment-originated data. To introduce the graph, the logical level of the graph database is presented. It is characterized by three features [11]:

1. Data and the database schema are represented by a graph.
2. Manipulation of data is expressed by graph transformations.
3. Integrity constraints (IC) enforce data consistency.

To reflect the first feature, the mathematical definition of a graph is introduced, followed by the presentation of the graph schema and the way of graph creation. Additionally, the data retrieving operations are described, which addresses the second feature. Data retrieving queries are expressed in Cypher language [12], the declarative query language for property graphs. The third feature is reflected by defining integrity constraints.

Because GRISERA framework is designed to store and retrieve data, the aim of validation is to check whether the data can be retrieved in a unified way. Therefore the successful data extraction is treated as the validation success. To validate the presented solution:

1. the generic queries in Cypher language are presented,
2. the GRISERA framework is implemented,
3. the two case studies are performed.

**Figure 1:**
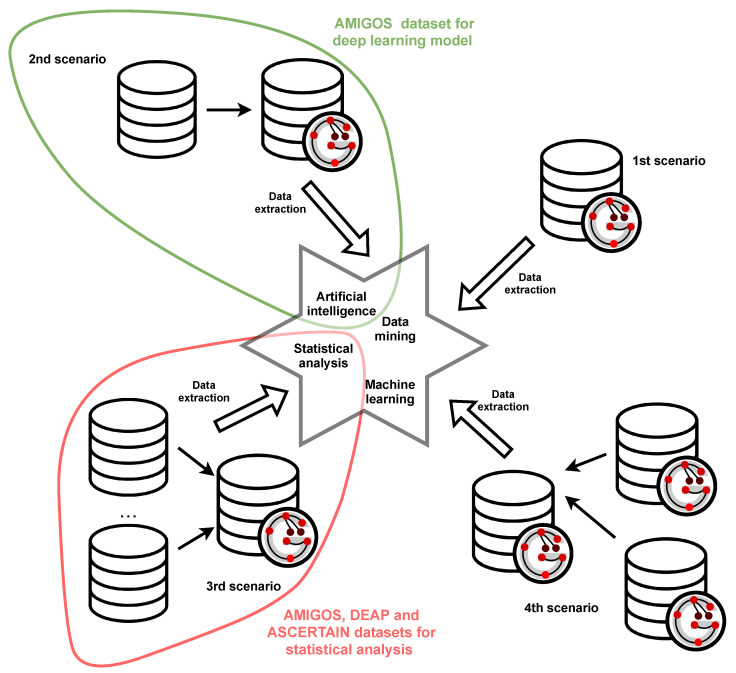
Application of GRISERA framework.

In Figure 1 four typical applications of the proposed framework are depicted:

1. experiment data creation with the GRISERA framework (1st scenario),
2. representing existing dataset in the GRISERA framework (2nd scenario),
3. integrating existing datasets into GRISERA framework (3rd scenario),
4. creating a new dataset in GRISERA framework from the existing datasets, also represented in GRISERA (4th scenario).

It is worth emphasizing that, according to the aim of the GRISERA and its applications the validation within the case studies is done by presenting graph creation and data retrieving. For data retrieving the list of competency questions is defined. Referring to the Figure 1 the extracted data can be used e.g., as a source for data mining, artificial intelligence, machine learning or statistical analysis, still, the results obtained from these methods are out of the scope of the validation process.

As stated earlier for validation purposes executed the following case studies were executed:

1. Usage of the GRISERA for unifying data from AMIGOS dataset [13] in order to build classification deep learning model distinguished in Figure 1 with green.
2. Integrating signals from various experiments originated from three datasets AMIGOS, ASCERTAIN [14] and DEAP [15] for statistical analysis depicted in Figure 1 with red.

The first case study covers the first and second scenarios. Although it refers to the integration of external datasets, it also includes the retrieval of data directly from the GRISERA framework. The second case study, on the other hand, covers the third and fourth scenarios. Proving the possibility of integrating data from external datasets, implies the possibility of integrating datasets already represented in the proposed framework.

Although the deep learning model and calculated statistics are mentioned for the completeness of case study presentation, they do not affect validity. Any aspects connected with applied deep learning models or performed statistics have no influence on the validation process.

## 4. Related Work

In the research conducted so far, a few databases with physiological data dedicated to emotion recognition experiments have been published, including DEAP, MAHNOB-HCI [16], DREAMER [17], HR-EEG4EMO [18], MPED [19] or Seal et al.’s EEG database [20]. However, they are all intended only to store data from experiments conducted by the authors. They do not offer the ability to add or integrate new signals originating from other studies. Moreover, the number of subjects included in each individual biosignal dataset dedicated to emotion recognition is insufficient to train high-quality machine learning models [21].

Siddharth et al. [22] proved that it is possible to use different biosignal datasets in order to train deep learning models for emotion classification purpose. In their research, they extracted features from four (DEAP, MAHNOB-HCI, AMIGOS and DREAMER) datasets. The proposed approach was the primary inspiration for ECG and GSR-based features for GRISERA framework, as it has led to better emotion classification results than reported by other studies [22]. Therefore, the integration of signals from different datasets seems to be a promising approach to solve the problem of insufficient data to train models.

Several studies have focused on the storage and integration of biosignals, although these are not related to emotion recognition [23,24,25,26]. For example, approach proposed by Kokkinaki et al. [27] is built on the definition of a global ontology that manipulates the source similarities and differences and thus creates mappings or enhances its structure. They also introduced the ROISES framework, for defining content-based queries against various biosignal datasets. However, their proposal is limited only to electrocardiogram biosignals.

Some proposals were based on using NoSQL databases to store signals. Carreiras et al. described a solution based on Hierarchical Data Format (HDF5) and MongoDB to store biosignal data in structured form. This proposal was based on an analysis of various database systems and file formats and their requirements and implementation capabilities in terms of the nature of biosignals [28].

From the analysis of available concepts of storing and integrating biosignals, a graph-based approach seems to be the most promising from our point of view. Graph representation allows you to capture relationships between biosignal sensors. A node can represent a single sensor and an edge between nodes can present information such as the correlation between nodes, e.g., how signals captured by one sensor activate other regions. In the initial stages of the research, we were looking for scientific papers related purely to the graph representation of biosignals.

Abdulla Shahab et al. [29] proposed the construction of the graph representation of EEG signals. Each of the channels of the EEG signals was partitioned into epochs of a specified length. Then signal nodes were connected based on a predefined correlation coefficient threshold. However, during our analysis, this approach failed to capture correlations between individual brain areas. What is more, using this approach we were not able to save signal values, thus valuable data was lost. This approach was mainly presenting how to construct an adjacency matrix with a predefined threshold, for sleep stages analysis, and presented a graph-based data structure that did not fulfil the function of storing and processing data.

On the other hand, Huang et al. [30] present a graph representation focused on capturing connectomes—the neural connections of the brain. While the model was based on Magnetic Resonance Imaging (MRI) data, it demonstrated the importance of capturing the particular correlations between brain areas. Such networks have been demonstrated to be correlated with behavioural measures and used in predictive modelling [31]. However, this approach did not present on the graph the changes in the signal over time, but only presented an image of the brain from one moment.

Later, we started to follow the example of works from graph signal processing, not necessarily limited to the topic of biosignals only. The paper of Sandryhaila et al. [4] describes graph representations of signals that served as a basis of all further work within the field of Graph Signal Processing (GSP). An example that we followed in this work was the graph representation of sensors measuring the weather conditions located across the United States. In this example, each graph node represents a sensor, storing the measured temperature and edges connecting nodes that are closely located to each other. For each timestamp, such a graph is created and then they are combined with each other through the Kronecker product, which allows for the analysis of changes in signal values over time. We found that representation universal and applicable to biosensors, where each node is a sensor storing measured signal values and edges represent correlation levels between nodes. As our goal is not only to store raw data but also process it and extract features for further analysis or machine learning purposes, we used node attributes to also store pre-computed values from the raw signal.

The research of Lotte, Fabien, et al. [32] proved to be pivotal when it came to the selection and extraction of EEG features that would be later used in the testing of the model’s efficacy. What is more, extracting features from the raw signal data and applying this expert knowledge can improve the accuracy of machine learning models. As graph databases are schema-less it is always possible to add new attributes such as pre-computed features to nodes and edges which additionally shows the legitimacy of choosing a graph database for biosignal modelling.

An analysis of available solutions and proposals revealed the need to integrate experiment originated data for various experiments and coherently store them for training machine learning models for emotion recognition [21]. In addition, the graph representation was selected for further work as allowing the best representation of the relationships between the signals.

## 5. Graph Representation

The unified graph representation for biosignals, emotional states and contextual information is the heart of the GRISERA framework. This representation is based on the two main assumptions:

- everything is represented as a graph and
- contextual information contains up-to-date information.

**Figure 2:**
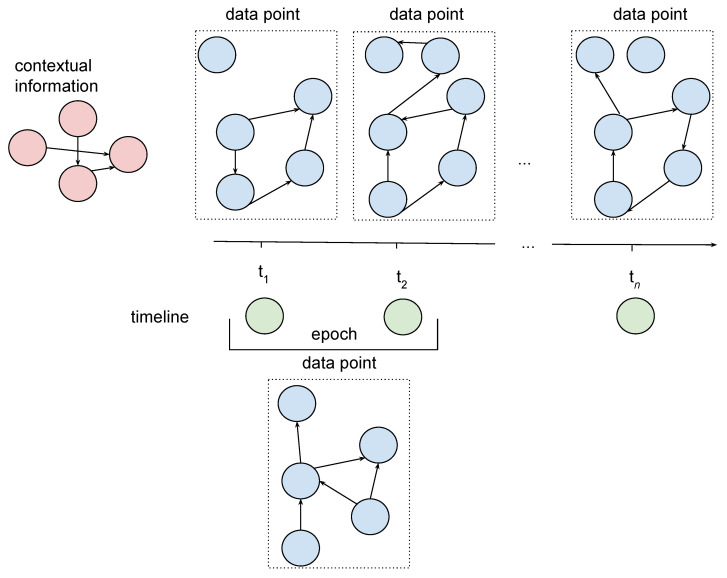
The idea of graph representation.

The idea is depicted in Figure 2. Each piece of information stored in the graph is assigned to one of the three groups: contextual information (red nodes), timeline (green nodes) and actual biosignal data or emotional states (blue nodes). This colouring rule is applied in all figures representing graphs.

The red nodes represent contextual information, including details about conducted experiment and participants taking part in it. Moreover, they contain information about channels that are the source of biosignals, as well as about recorded measures, including emotional states. Meanwhile, edges carry information about the relationships between these notions. An example of contextual information could be: watching movies as an activity performed during the experiment, EEG or GSR as channels used to observe participants, a 16-channels EEG system as a standard EEG recording system or peak count as a measured value for a GSR channel.

The timeline is presented in green and is understood as a set of time moments or epochs, which are periods of time with a beginning and ending at specified time moments.

A single data point, depicted as a set of blue nodes outlined with a dotted line, carries information about values of all measures, acquired from all integrated signals, obtained from all available channels or values representing emotional states. The data point can refer to a single moment of time (in Figure 2, data points above the timeline) or to the specified epoch (in Figure 2, a single data point below timeline). Signals with data points referring to a single moment of time are further called timestamp signals, while signals with data points referring to an epoch are further called epoch signals. Edges connecting nodes within data points imply that there exists a dependency of some nature between the values stored in those nodes.

Such construction allows one to:

- represent diverse biosignals, both of timestamp signals or epoch signals type,
- cope with missing values—some nodes of data points or node properties may not contain information about the value or new values can be calculated according to the widely known approaches for missing values; the strategy depends on the requirements provided by applicational use of integrated experiment originating data,
- solve the problem of various frequencies—presented model can store timestamp signals with different frequencies and analogically for epoch signals it is possible to represent them with various epoch length; it is also possible to build epoch signals based on original values being timestamp signals, what is important in some applications e.g., deep learning or statistical analysis where the epoch length should be constant for all signals.

### 5.1. Graph Definition

Graph definition is based on the definitions of the property graph model introduced by Angles et al. [33,34]. It is assumed that having set X, $SET^{+}\left(X\right)$ is the set of all finite subsets of *X*, excluding the empty set.

**Definition** **1** **(Labeled property graph).** *A labeled property graph G is a tuple (V, E, ρ, $\lambda_{1}$, $\lambda_{2}$, σ), where:*

*(1) V is a finite set of vertices (or nodes).*

*(2) E is a finite set of edges such that V and E have no elements in common.*

*(3) ρ: E → ( V × V ) is total function.*

*(4) $\lambda_{1}$: V →$SET^{+}$(VerLabs) is a total function with VerLabs a set of labels possible to assign to vertices.*

*(5) $\lambda_{2}$: E → EdgeLabs is a total function with EdgeLabs a set of labels possible to assign to edges.*

*(6) σ: (V ∪ E)× Prop → Val is a partial function with Prop a set of properties and Val a set of values.*

In Definiton 1 each edge is directed from one node to the other. Each node is associated with one or more labels and each edge is associated with a single label. Each node and edge is also associated with at most one value of each property (albeit the value can be a simple value or a list). The *Prop* set is not finite. The current version of the graph defines the subset of *Prop* and allows for new user-defined elements. The elements of the *VerLabs*, *EdgeLabs* and the subset of *Prop*, with the semantics of each element, are presented in Appendix A (respectively in Table A1, Table A2 and Table A3).

**Figure 3:**
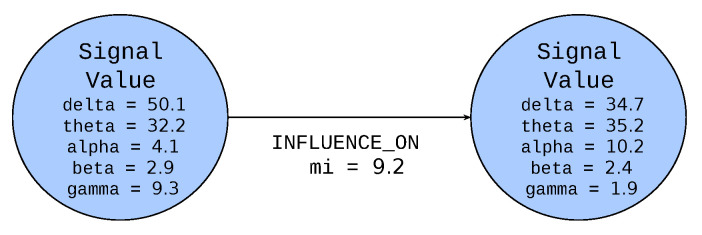
Node notation.

The Figure 3 presents the notation for the node and edge. The circle represents a node inside of which the specified label is defined (Signal Value in the example). The node label (an element of *VerLabs* set) is represented as a noun or a noun expression in uppercase monospace font. Below the label the list of properties represented as nouns or nouns expressions in lowercase monospace font (delta, theta, alpha, beta and gamma in the example) are depicted. The values (elements of *Val* set) are written using serif italic font (in the example float values). The line represents an edge and the arrow indicates the direction of the edge. The labels of edges (an element of EdgeLabs) are represented as capitalized verbs or verb expressions. Below the edge label, the list of properties and their values is depicted in the same manner as for nodes. As it was mentioned previously the colour of the node defines the group, to which the node belongs.

### 5.2. Graph Schema Proposal

**Figure 4:**
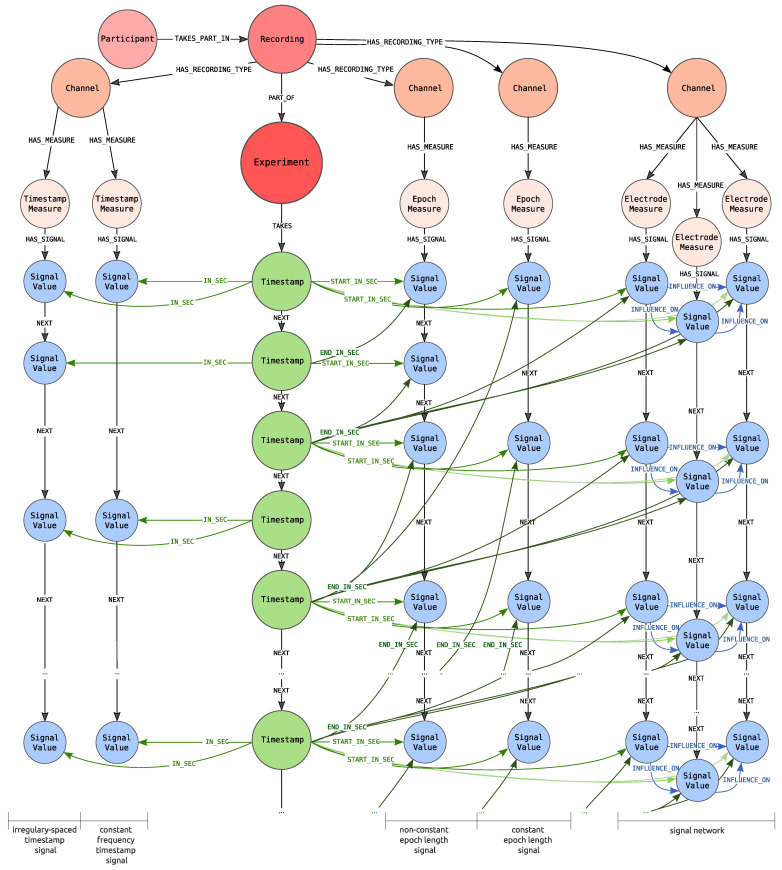
Proposed graph database schema.

The central point of the proposed graph, shown in Figure 4, is an Experiment node (coloured in red) that stores information about the conducted experiment, e.g., activity performed by the participants, the place where the experiment took place like room and layout. The red colour of the Experiment carries information that this node represents contextual information in an analogy to Figure 2 (blue colour corresponds to the data points and green to the timeline).

The Recording node, connected to the Experiment node via PART_OF edge, represents the participation of the specified person, denoted as Participant, within the specified Experiment. There can be more than one Recording nodes pointing to the same experiment. It allows modelling the participation of a single participant within more than one Experiment as well as experiments in which more than one person participates.

The Experiment node is connected with the TAKES edge to one Timestamp node. By Timestamp it is meant the distance from the beginning of the recording expressed in milliseconds (it is assumed that there is no need for the smaller unit). Each experiment can only have a single timeline. In practice, this means that all of the signals recorded as a part of the same experiment have a common timeline. The graph representation of the timeline is constructed as a linked list of Timestamp nodes. Pairs of consecutive Timestamp nodes are connected by a NEXT edge.

Each Recording node in our graph can generate many signals, which are understood as channels with recorded measures and the series of signals values. Recording is connected to one or more Channel nodes with a HAS_RECORDING_TYPE edge. Channel node indicates that the Participant is observed via a specified channel from which signals of defined types are recorded. This node can also define the channel used to recognize participant emotions. It is worth noticing that the Channel represents the recorded channel for the specified Recording and not the channel in general.

Depending on the channel type, each node can be connected to one or more nodes representing measures (Measure nodes), due to the fact that from a single channel several signals, each referring to the particular measures, can be obtained. Two types of measures are identified: Timestamp Measure for timestamp signals (left of the timeline in Figure 4) and Epoch Measure for epoch signals (right of the timeline). Timestamp Measure is used when measures determine values at a specific point in time, e.g., body temperature. On the other hand, the Epoch Measure is used for the values that are settled or calculated in some way for the whole epoch. A good example is the number of GSR peaks, which can be calculated only over a specified period of time. There is also the special case of epoch measure i.e., Electrode Measure node, which allow distinguishing signals of the same type but different placement. These nodes define from which place of the body connected signal were recorded. For example, ECG may have two types of electrodes including left and right sensors, whereas the EEG may have 14 types that were described by the international 10–20 system. What is even more important Electrode Measure is used when there is a need to represent influence between the two values of the two different electrode measures, as long as the electrode measures concern the same channel.

Finally, Signal Value nodes store information about values of measurements. Each Signal Value relates to timeline’s Timestamp node with IN_SEC or with START_IN_SEC and END_IN_SEC edges—depending on the type of signal. For timestamp signal IN_SEC edge is used and points at the measurement time. For epoch measure START_IN_SEC edge marks the start of the epoch. The end of the epoch is determined by the END_IN_SEC edge.

In addition, each Signal Value node is connected to its successor with the NEXT edge. The Measure node is connected with the HAS_SIGNAL edge with the first node within the series of signal values (the one which is not the object of the NEXT role). This construction is ready for storing the information about missing values represented as nodes with no values set. The same feature can be used to fill the missing value with appropriate calculations.

When Signal Value nodes represent Electrode Measure values, they can be related via the INFLUENCE_ON edge, representing the influence of one value on the other one.

Each of these nodes can additionally have properties. For the nodes describing contextual information the properties are used to describe metadata information of the node. For instance, the Participant node has properties describing the given person such as user_id, gender or age. For Measure node the signal_id property is introduced to identify signals. In case of Timestamp measure and Epoch measure additionally the properties name, datatype and range are set. The Electrode measure has the property name identifying the electrode, while the Channel defines properties describing the channel like type or recording standard.

For the nodes describing data points i.e., Signal value nodes the properties contain measured values. In case the Signal value is represented by the Timestamp Measure, the property value is defined. An analogous construction is applied for Epoch Measure when the value is settled—not calculated. Otherwise, both the calculated values as well as the original ones are represented. The original values are stored within data property that is an array of values, while the calculated values are dependent on the channel (e.g., the different values are calculated for EEG and ECG channels).

Likewise, for the Timestamp node, only the value representing the distance from the beginning of the recording is stored.

#### 5.2.1. Signal Representation for Timestamp and Epoch Measures

In graph databases, there are a few ways in which time-series data can be modelled [35]. The most common approach is to model each measurement as a separate node and connect it with measurement nodes immediately before and after it through the NEXT edge [36]. The first measurement node in the series is connected with a parent node, that describes what measurements they represent. According to this assumption, the signals representing timestamp measures and epoch measures are designed.

According to the definition, timestamp signals are the signals, which values always correspond to the specific time moment. These signals can have constant frequency, e.g., participant temperature measured every second. In such a case the Timestamp Measure has the name, type and range properties.

This type of signal can also be used to represent emotional states obtained from emotion recognition methods. The example can be emotional states generated from Face Reader software [37] when the constant frequency is settled. There are no premises allowing us to assume that the recognized emotional state concerns the whole epoch. If the temperature would be measured irregularly we would be dealing with irregularly spaced timestamp signal.

On the other hand, epoch signals are the ones, which values relate to the whole epoch. This kind of signals is very useful to represent emotions. Let’s take as an example data collected in the process of labelling facial expression videos, during which annotators label emotional intensity using the two-dimensional Russell’s Circumplex Model of Affect [38]. Each recording is divided into segments and each segment is annotated with specific measures (reflecting emotional states of participants). We can notice that the length of the segments can be constant (then we deal with constant epoch length signal) or not (then we deal with variable epoch length signal).

The other type of signal is calculated value epoch signal. It means that we have the original data stored in data property and new values are calculated. For each newly calculated value, the appropriate property is created. This type of signal is especially needed when we have a timestamp signal and want to transform it to an epoch signal or we have an epoch signal and want to create a new epoch signal with a bigger epoch length. This is often needed in the process of signal integration when all signals must have a unified epoch length. The example can be the average temperature for the whole epoch, calculated from the timestamp signal. For such signals, the framework provides the solution named calculators. The aim of the calculators is to calculate new values from original ones e.g., minimum, maximum or average. The calculators are further discussed in Section 6.

#### 5.2.2. Signal Representation for Electrode Measures

Throughout the analysis of biosignals it became clear that some of them—especially bioelectrical ones—may be perceived informally as complex signals, being a set of, correlated in some way, signals originated from separate electrodes. These complex signals are further called signal network. Each signal obtained from a single electrode can be seen as a calculated value epoch signal with constant epoch length. The characteristic of signals obtained from electrode may demand some prepossessing of original values before calculating the new ones (like filtering or resampling). Additionally, the values from one electrode in some way can have an influence on values from other electrodes, which is especially the case for the EEG channel [39]. All these features caused that the new type of measure Electrode Measure was introduced. It is important to notice that all signals within the signal network have exactly the same epoch length.

**Figure 5:**
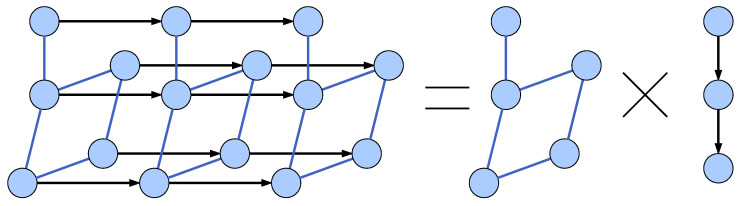
Sensor network measurements as a Cartesian product of sensor network and time series graphs.

Signal network is understood as a sensor network measurements graph [4] implemented as a combination of a sensor network graph with a time-series graph via a Cartesian product as depicted in Figure 5, consistently with the colour scheme in Figure 4. Blue edges represent the correlation between Signal Value nodes. A time-series graph is understood as a calculated value epoch signal with constant epoch length. The original model used a strong product as opposed to a Cartesian product. This resulted in edges connecting pairs of nodes representing signals from different electrodes and moments in time. While we decided to simplify the model we understand that there may be merit to calculating correlations between these nodes and it may be introduced in the next version of GRISERA framework.

The preprocessing of bioelectrical signals often consists of reducing noise by applying a band-pass filter (BPF) and then resampling the signal. The specific cutoff frequencies used for the BPF and resampling configuration are dependent on the channel and are based on previous research within the field of machine learning [22].

The signal network, for each type of channel, has its own set of calculated values selected. These values are identified for each channel separately. Within this paper, the representation of the signal network is analyzed for three channels (GSR, ECG and EEG), although this set can be extended at any moment just by providing new calculators (for the new channels or new calculated values). This also applies to property calculations for graph edges.

#### 5.2.3. Galvanic Skin Response—GSR

Galvanic Skin Response is understood as a change in the electrical resistance of the skin that is a physiochemical response to emotional arousal which increases sympathetic nervous system activity [40].

GSR calculated values are based on the change of the skin conductance [41]. A sudden increase in signal value is often called a GSR peak. Its extraction is based on the approach presented by H. Gamboa [42]. All calculated values corresponding to detected peaks are represented as maximum (property GSR_max), minimum (property GSR_min) and average (property GSR_avg) values. In addition, the number of occurrences of peaks is calculated (property no_of_peaks). The signal network for GSR consists of exactly one Electrode Measure named GSR.

#### 5.2.4. Electrocardiography—ECG

Electrocardiography allows the recording of changes in the electrical potential that occur during heart contraction, used especially in the diagnosis of cardiac disorders [40].

The main calculated value extracted from the ECG signal is the heart rate variability—HRV (property HRV). It represents the variability in time taken between consecutive heartbeats—the interbeat intervals or R-R intervals. The name of these intervals comes from R-peaks—the distinct spikes are seen on an ECG line. The specific indices of these peaks are extracted following the approach by Engelse and Zeelenberg with changes made by Lourenco et al. [43]. The signal network for ECG typically consists of two Electrode Measures named left and right. The calculated properties contain the set of aggregated values for HRV and interbeat intervals.

#### 5.2.5. Electroencephalography—EEG

Electroencephalography is a brainwave detection and recording device [40]. There are more than one international systems defining electrodes and their placement (e.g., 10–20, 10–10 or 10–5). The difference in representation is connected with the number and names of Electrode Measure nodes. For the EEG signal, the band power features—also known as the Power Spectral Density (PSD)—are calculated. They represent the power of the signal within functionally distinct frequency ranges. These ranges are the delta (0.5–4 Hz), theta (4–8 Hz), alpha (8–12 Hz), beta (12–30 Hz), and gamma (30–100 Hz) channels. Decomposing the signal into individual frequency ranges is done using the Multi-Taper Method (MTM) developed by Thomson [44]. This method is used as opposed to the classic Fourier Transform because it returns more accurate results a non-stationary signal. The spectral content of the EEG tends to change over time, so this aspect of the algorithm improves the accuracy of the results. Following the proposed methodology, the Slepian sequence is used as tapers [45]. A weighted average is also used to compensate for the energy loss of higher-order tapers [46].

PSD is chosen as one of the extracted features because it strongly correlates with emotional responses. Increased Gamma waves can be a sign of excitement, Beta waves signify attentiveness and focus, while Alpha waves mean joy and relaxation. Both Theta and Delta waves correlate with sadness and shock, but Theta frequencies can additionally signify anger.

Mutual information (MI), is used to represent correspondence between nodes of EEG electrode signals. Mutual information *I(X;Y)* of two discrete random variables *X* and *Y* is the embodiment of their mutual dependence. The relationship between MI and conditional entropy *H(Y|X)* was used to calculate its value, like so

(1) I(X;Y)=H(Y)−H(Y|X)

where *H(Y)* represents the entropy of discrete random variable *Y*. The entropy, as a probability of observing a certain outcome x of a discrete random variable *X*, was estimated using maximum likelihood estimation. This feature is stored as a property mi for INFLUENCE_ON edge between pairs of EEG Signal Value nodes.

### 5.3. Integrity Constraints

Integrity constraints are introduced for node property uniqueness and mandatory properties in the Cypher language.

A node property uniqueness IC states that for the set of nodes with the specified label the specified property must be unique. In the Listing 1 it is stated that for the nodes with Experiment label (expressed in ON phrase) the value of the name property is unique (expressed after ASSERT phrase).

**Listing 1.** Experiment name node property uniqueness ICs.
CREATE CONSTRAINT ON (n:Experiment) ASSERT (n.name) IS UNIQUE;


For the GRISERA graph also three other node property uniqueness constraints are defined. The first one states that the value of user_id property must be unique within nodes with Participant label. The second one states that value of the Timestamp nodes is unique within this set. The last one is analogical but for signal_id of the Measure nodes.

Mandatory properties ICs can be defined both for nodes and edges. This type of IC states that for nodes or properties with the specified label, the value of the specified property must exist—cannot be null. In the Listing 2 it is stated that for nodes with Channel label the not null value of the property type must be defined (expressed in the EXISTS phrase). The full list of mandatory properties ICs together with possible values of properties is defined in Appendix B.

**Listing 2.** Mandatory properties ICs.
CREATE CONSTRAINT ON (n:Channel) ASSERT EXISTS (n.type)


## 6. Graph Database Creation

The GRISERA graph is created with the use of Cypher query language. The first two Algorithms 1 and 2 present the elementary graph creation operations i.e., node and edge creation respectively. Algorithm 1 is straightforward—it takes the subset of labels, which the node should have been assigned, the set of property-value pairs to be attached to the node, creates it and returns the node identity.
**Algorithm 1:** Node creation** Input**:  *S* ← subset of *VerLabs*; *P* ← $\left\{p_{1}:v_{1},p_{2}:v_{2},\ldots ,p_{n}:v_{n}\right\}$;**1** labels ← *S***2** properties ← *P***3** query ← “*CREATE (n:labels properties) RETURN id(n)*;”**4** run query

Algorithm 2 creates a labelled edge between the two nodes given as an input.
**Algorithm 2:** Edge creation** Input**:  $s_{1}$ ← node; $s_{2}$ ← node; *l*← element of *EdgeLabs*; **1** query ← “$MATCH\left(s_{1}\right),\;\left(s_{2}\right)\;WHERE\;id\left(s_{1}\right)\;=s_{1}.id\;AND\;id\left(s_{2}\right)=s_{2}.id\;CREATE\;\left(s_{1}\right)$-[:*l*]  → $\left(s_{2}\right)$;”**2** run query

The graph creation is presented by describing the steps of creating the Recording node (Algorithm 3), signals’ values (Algorithm 4) and timestamps corresponding to them (Algorithm 5 for timestamp signals and Algorithm 6 for epoch signals).
**Algorithm 3:** Recording creation
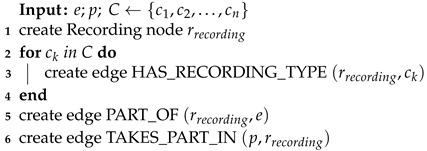


The input of Algorithm 3, creating the Recording, is: *e*—the node representing the experiment, *p*—the node representing the participant being recorded and *C*—the set of nodes representing channels, the participant is observed by. The algorithm first creates the Recording node and then connects it with all input Channel nodes, Experiment node and Participant node.

**Figure 6:**
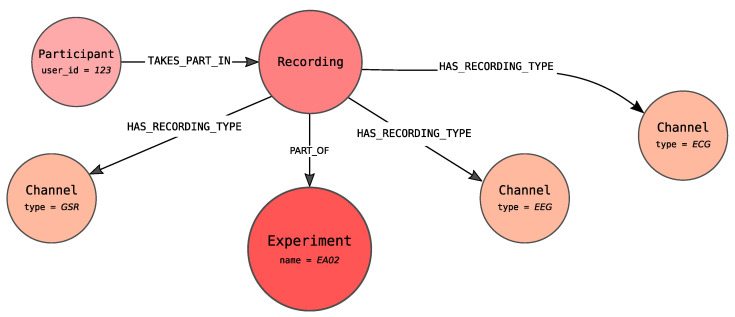
Contextual information for the exemplary Recording node.

The result of Algorithm 3 for the experiment exp1, in which one participant identified with 123 number and observed with three channels (GSR, EEG and ECG), is depicted in Figure 6.
**Algorithm 4:** Signals’ values creation
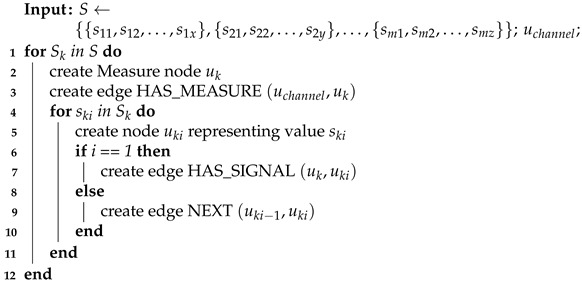


The process of creating signals in the graph database may vary a bit, depending on various parameters like frequency or epoch length, but the general form of the algorithm remains the same. Firstly, the signals’ values are imported and after that connected with the timeline.

The Algorithm 4 takes as an input the list of lists of values for various signals generated by the observation of the specific channel and creates for each list the corresponding Measure node with appropriate label (Timestamp Measure, Epoch Measure or Electrode Measure) and properties. This Measure node is also connected with the signal values of the same list.

Connection with timeline nodes varies depending on the signal type. For timestamp signal (Algorithm 5) only one edge (IN_SEC) for each value is created—the one reflecting measure time. In the case of epoch signal (Algorithm 6) with each value, a pair of edges is created— representing the start and end of the epoch. Referring to the Algorithm 5 in Algorithm 6 the if structure used to get an existing Timestamp node or insert a new node into the Timestamp linked-list structure using NEXT edges has been shortened to “create or get node” for readability.

**Figure 7:**
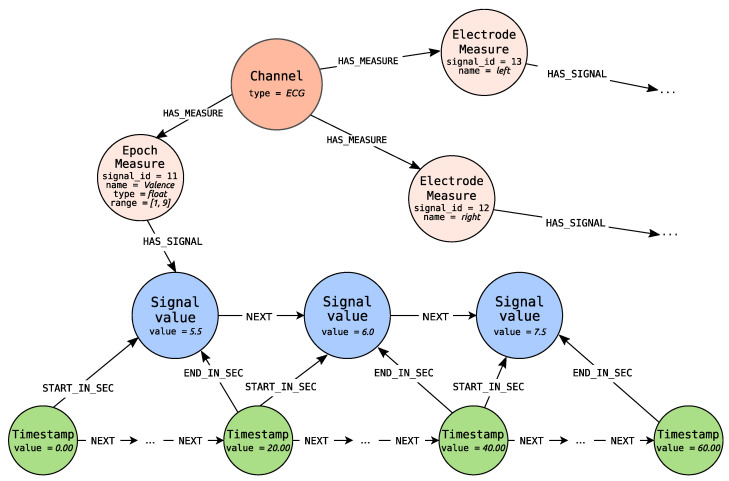
Constant epoch-length signal representing measured valence values.

In Figure 7 the exemplary constant epoch length signal for ECG channel, generated by Algorithms 4 and 6, is depicted. Algorithm 4 takes as an input three lists of signal values (for right and left Electrode Measure and for Epoch Measure representing recognized from ECG valence estimates). The exemplary signal is presented for Epoch Measure. The epoch length is set to 20 s. Epoch Measure has values set for three properties name, type and range. The values are represented as float values in the range from 1 to 9 inclusive. Signal Value nodes carry information about valence values, settled for the whole epoch (the specific 20 s) in the value property. The value property of the first Timestamp node is equal to 0ms, which denotes that it is the starting point for the recording. The next signal values are measured every 20 s (that is 20,000 milliseconds). Some Timestamp nodes are omitted because other timestamps can exist for the need of other signal representation. The example presents the signal, which values are settled and not calculated—source value epoch signal.
**Algorithm 5:** Assigning signal values to timestamps
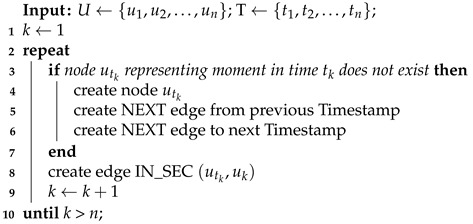


**Algorithm 6:** Assigning signal values to epochs

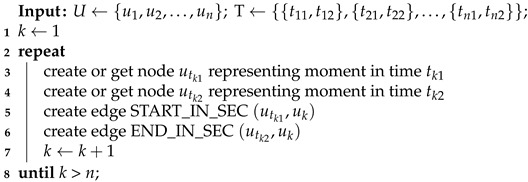



In the case of epoch signal, once it has been imported into the database, descriptive values can be extracted from it. These can either be calculated based on single epochs or represent a relationship between two epochs. In the case of the former, the matter of extracting said values is as simple as applying a chosen function to the arrays of numerical values stored in the signal value nodes and then assigning the results as node parameters. The latter is shown in Algorithm 7. It takes *m* signals of length *n* for every Measure from a single Channel—denoted as *U*–and an optional threshold value μ.
**Algorithm 7:** Signal attribute calculation
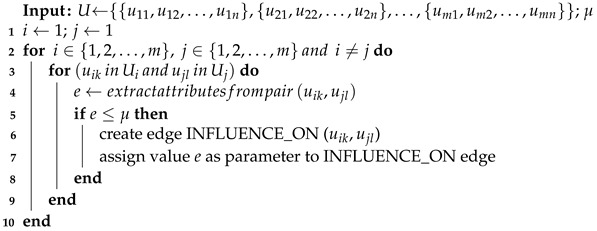


### Memory Efficiency

Memory efficiency will be described in terms of nodes and edges created during the importing and feature extraction process. We assume a signal with a constant frequency of *f* (Hz) and a length of *t* (seconds) consisting of *m* time series. Creating an epoched representation of this signal using GRISERA will result in the following number of nodes:

(2) $$\begin{array}{cccc} \; & s=\lfloor \frac{ft}{t_{e}}\rfloor \; & \; & \mathrm{nodes}\;\mathrm{per}\;\mathrm{signal}\;\mathrm{measure}\\ \; & S=ms=m\lfloor \frac{ft}{t_{e}}\rfloor \; & \; & \mathrm{total}\;\mathrm{nodes} \end{array}$$

where $t_{e}$ is the length of the epoch in seconds. The framework also allows for the use of the sliding window method, which generates a different number of nodes. Given a length of the window *w* and of overlap *o* we arrive at:

(3) $$\begin{array}{cccc} \; & s=\lfloor \frac{ft-w}{w-o}\rfloor \; & \; & \mathrm{nodes}\;\mathrm{per}\;\mathrm{signal}\;\mathrm{measure}\\ \; & S=ms=m\lfloor \frac{ft-w}{w-o}\rfloor \; & \; & \mathrm{total}\;\mathrm{nodes} \end{array}$$

During feature extraction, edges are created between pairs of signal nodes belonging to the same epoch. While the creation of these edges may depend on a threshold being met by the calculated value, it can be assumed that all edges possible edges are created. Under this assumption, a complete graph is created from signal nodes within every epoch of a signal.

(4) $$\begin{array}{cccc} \; & r=\frac{m(m-1)}{2}\; & \; & \mathrm{edges}\;\mathrm{per}\;\mathrm{epoch}\\ \; & R=sr=s\frac{m(m-1)}{2}\; & \; & \mathrm{total}\;\mathrm{edges}\\ \; & R_{e}=\left\lfloor \frac{ft}{t_{e}}\right\rfloor \frac{m(m-1)}{2}\; & \; & \mathrm{total}\;\mathrm{edges}\;\mathrm{for}\;\mathrm{given}\;\mathrm{epoched}\;\mathrm{signal}\\ \; & R_{s}=\left\lfloor \frac{ft-w}{w-o}\right\rfloor \frac{m(m-1)}{2}\; & \; & \mathrm{total}\;\mathrm{edges}\;\mathrm{for}\;\mathrm{given}\;\mathrm{the}\;\mathrm{sliding}\;\mathrm{window}\;\mathrm{method} \end{array}$$

## 7. Data Retrieval

The presentation of data retrieval is done for the set of defined competency questions, which are further expressed generically in the Cypher language. Competency questions are divided into two subsets. The first subset of the competency questions does not demand the unification of signals to the same timestamp frequency or epoch length. This subset contains questions regarding experiments and signals search as well as data extraction for one signal. The second subset contains questions for data retrieval for signals unified to the same timestamp frequency or epoch length. This subset contains questions allowing to retrieve multivariate time series built from data stored in various signals or retrieve the specified set of signal values.

Competency questions for experiments or signals search and data retrieval for one signal:

1. CQ1—What experiments contain signals for the specified measures, which values are obtained through observation of the specified channels?
2. CQ2—What signals relate to the specified measure, which values are obtained through observation of the specified channels?
3. CQ3—What is the times series for the specified signal?

Competency questions retrieving multivariate time series:

1. CQ4—What is the multivariate time series for the specified signals?
2. CQ5—What is the multivariate time series for the specified signals containing only these signal values which fulfil the specified conditions (values of some measures must conform to the specified requirements)?

The first two competency questions, expressed in Cypher language in Listings 3 and 4 refer to contextual data searching and consist of the basic Cypher structure i.e., the pattern to be searched, expressed in MATCH clause. In the CQ1 this is a pattern defining nodes with label Experiment being connected with Recording node (not explicitly stated as the subject of PART_OF edge can be Recording node only), which is further connected with the Channel node with the specified type, depicted as a parameter <type>. Next, the channel is connected with the node being a measure of any type (each has the name property obligatory and each can be an object of HAS_MEASURE edge). The second parameter <name> is the name of the measure.

**Listing 3.** Generic Cypher CQ1.MATCH (e:Experiment)<-[:PART_OF]-()-[:HAS_RECORDING_TYPE]->(c:Channel {type:<type>})-[:HAS_MEASURE]->(m {name:<name>}) 
RETURN e


**Listing 4.** Generic Cypher CQ2.MATCH (c:Channel {type:<type>})-[:HAS_MEASURE]->(m {name:<name>}) RETURN m

In the CQ2 MATCH clause defines just Channel nodes connected with measure nodes. The parameters of the second competency question are analogical as for the first one.

The aim of the CQ3 depicted in Listing 5 is to provide a unified way of extracting time series for the specified signal (identified with the <signal_id>). The pattern in MATCH phrase allows to find all Signal Value nodes for the specified signal and timestamps connected with them (measure timestamp for timestamp signal and timestamp starting the epoch for the epoch signal—also for the one obtained from the electrode). OPTIONAL MATCH phrase allows retrieving the timestamp ending the epoch. This phrase can be omitted when the values of the timestamp signal are retrieved, otherwise ending timestamps are null values.

**Listing 5.** Generic Cypher CQ3.
MATCH (m {signal_id:<signal_id>})-[:HAS_SIGNAL]->(s1_root:SignalValue)-[:NEXT*0..]->(s1:SignalValue)<-[:IN_SEC]-(startTimestamp:Timestamp)

OPTIONAL MATCH (s1:SignalValue)<-[:END_IN_SEC]-(endTimestamp:Timestamp)

RETURN startTimestamp.value, endTimestamp.value, s1.value


The CQ4, depicted in Listing 6, when retrieving the multivariate time series, adds in relation to CQ3, the list of OPTIONAL MATCH phrases. These additional phrases allow retrieving signal values for the set of various additional measures (m2, ..., mn). Each measure reflects one column in the resulted data.

**Listing 6.** Generic Cypher CQ4.
MATCH (m1 {signal_id:<signal_id#1>})-[:HAS_SIGNAL]->(s1_root:SignalValue)-[:NEXT*0..]->(s1:SignalValue)<-[:IN_SEC]-(startTimestamp:Timestamp)

OPTIONAL MATCH (s1:SignalValue)<-[:END_IN_SEC]-(endTimestamp:Timestamp)

OPTIONAL MATCH (m2 {signal_id:<signal_id#2>})-[:HAS_SIGNAL]->(s2_root:SignalValue)-[:NEXT]->(s2:SignalValue)<-[:IN_SEC]-(startTimestamp)

...

OPTIONAL MATCH (mn {signal_id:<signal_id#n>})-[:HAS_SIGNAL]->(sn_root:SignalValue)-[:NEXT]->(sn:SignalValue)<-[:IN_SEC]-(startTimestamp)

RETURN startTimestamp.value, endTimestamp.value, s1.value, s2.value, ...,

sn.value


The last competency question—CQ5, presented in Listing 7, allows defining restrictions for the specified measures (r1, ..., ri). The retrieved data are multivariate time series, but only these signal values are returned which meet the specified restrictions. The specified restrictions are defined in the MATCH phrase (after the first comma) and WHERE clause.

**Listing 7.** Generic Cypher CQ5.
MATCH (m1 {signal_id:<signal_id#1>})-[:HAS_SIGNAL]->(s1_root:SignalValue)-[:NEXT*0..]->(s1:SignalValue)<-[:IN_SEC]-(startTimestamp:Timestamp),

(r1 {signal_id:<restricted_signal_id#1>})-[:HAS_SIGNAL]->(rs1_root:SignalValue)-[:NEXT*0..]->(rs1:SignalValue)<-[:IN_SEC]-(startTimestamp),

...

(ri {signal_id:<restricted_signal_id#i>})-[:HAS_SIGNAL]->(rsi_root:SignalValue)-[:NEXT*0..]->(rsi:SignalValue)<-[:IN_SEC]-(startTimestamp),

WHERE <restriction_1> AND ... <restriction_i>

OPTIONAL MATCH (s1:SignalValue)<-[:END_IN_SEC]-(endTimestamp:Timestamp)

OPTIONAL MATCH OPTIONAL MATCH (m2 {signal_id:<signal_id#2>})-[:HAS_SIGNAL]->(s2_root:SignalValue)-[:NEXT]->(s2:SignalValue)<-[:IN_SEC]-(startTimestamp)

...

OPTIONAL MATCH (mn {signal_id:<signal_id#n>})-[:HAS_SIGNAL]->(sn_root:SignalValue)-[:NEXT]->(sn:SignalValue)<-[:IN_SEC]-(startTimestamp)

RETURN startTimestamp.value, endTimestamp.value, s1.value, s2.value,

s3.value, ..., sn.value


## 8. Validation

For validation purposes, the GRISERA framework was implemented (Section 8.1) and then applied to two scenarios. In the first one, data from AMIGOS dataset were integrated in GRISERA and then used to train a classification deep learning model (Section 8.3). In the second scenario data for multiple datasets AMIGOS, ASCERTAIN and DEAP were integrated within GRISERA framework (Section 8.4).

### 8.1. Implementation

**Figure 8:**
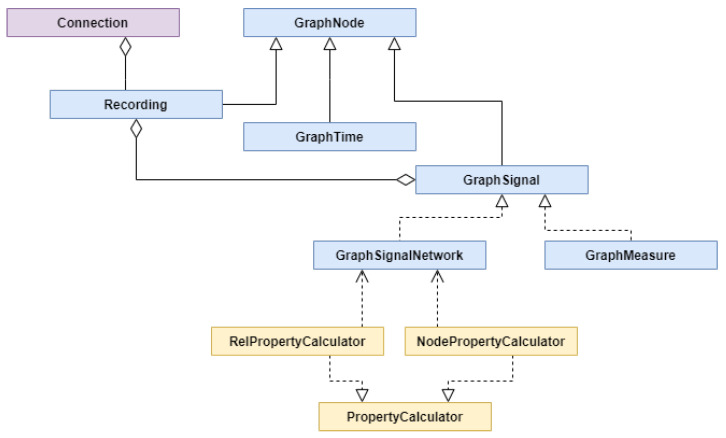
UML class diagram.

The task of creating the graph representation of experiment originating data was implemented as scripts that performed the Extract-Transform-Load (ETL) process. They were implemented in Python language using data manipulation libraries like Pandas and Numpy. First, we extracted the data from the sources, and after transformations, moved them to internal structures that represent parts of the graph, as presented in Figure 8 in blue. Then the calculators, depicted in yellow, were used to enrich the signal network with newly calculated properties to prepare them for storage. Finally, all parts of the graph were stored in Neo4J graph database.

A Connection class (purple) was responsible for establishing a connection with Neo4J and checking the imported recordings list. It was used by each of the class representing parts of the graph. GraphNode was a representation of any node in the graph and it was identified by a unique ID that was granted by Neo4J. A Recording class inherited from GraphNode and represented a node of a recording for a participant in an experiment. It was connected with GraphTime that represented a timeline of an experiment and also was a GraphNode. The next important class was a GraphSignal that was directly connected with a Recording class. It represented a series of signal values. These signal values were represented by GraphMeasureSignal or GraphSignalNetwork classes. The first one represented a constant frequency timestamp signal or source value epoch signal with constant epoch length. The second one represented a signal network. For signal networks, there were two types of calculators implemented. The one RelPropertyCalculator was used to calculate relationships between signal values and the other one NodePropertyCalculator to calculate new values from original data. This model was expandable allowing to implement classes for other types of signals and to implement new calculators of both types.

According to Jim Webber (Chief Scientist at Neo4J), the disk size ratio required to store similar data in different no relational databases is 20x = 50y = 0.33z [47]. Where x = MongoDB, y = Cassandra and z = Neo4J. It means that storing graph data in Neo4J is much more efficient than storing it in Cassandra or MongoDB.

Neo4J uses fixed record lengths to persist data. In Table 1 we can see how much disk size is taken up by elements of the graph stored in Neo4J.

Based on the number of each element in the graph and the memory taken up by each of them, we could calculate the total disk size needed to store the whole graph.

### 8.2. Datasets Description

Three datasets that are available for research purposes were used for validation. The AMIGOS dataset consists of data gathered from 40 participants, it consists of 16 short videos (up to 2 min), four long videos (14–23 min) and it stores data from EEG, ECG and GSR signals [13]. The DEAP dataset has data taken from 32 participants, 40 1-minute length clips of music videos were used as a stimulus for the participants [15]. Finally, ASCERTAIN dataset contains experiment originated data for 58 users viewing affective videos along with EEG, ECG, GSR and facial activity data [14].

### 8.3. AMIGOS Dataset for Deep Learning Model

The first scenario shows that it was feasible to use the GRISERA framework to store experiment originating data and then retrieve it for the purpose of deep learning modelling. Data from the AMIGOS dataset were saved in a graph database. Then, a neural network used data retrieved from GRISERA for emotion classification based on values pre-computed from bioelectrical signals.

As an a priori task for the automatic emotion recognition is a selection of features [48] the following properties for each type of signal were calculated:

- for EEG signal: band power features and mutual information for all 14 electrodes, as it is described in Section 5.2.5.
- for ECG signal: heart rate variability and interbeat intervals values like the minimal value, maximal value, variance and mean for each epoch, as it is described in Section 5.2.4.
- for the GSR signal: GSR peaks as the minimal value, maximal value, and the average.

The AMIGOS data, with these pre-computed features, was saved to the graph database with the schema described in Section 5 and algorithms described in Section 6. The uniform and well-described schema allowed extracting the most important properties into the training set. For each 20-s epoch (that was the frequency of annotations) extraction of a feature vector with 181 values was made and attached to annotations representing emotions. Out of those values 90 was calculated on individual signal nodes—single epochs—and included five bandpower values from every of the 14 EEG electrodes, eight values relating to heart rate and heart rate variability from the two ECG electrodes and four GSR values. The remaining 91 values came from edges between EEG nodes and denoted the mutual information between pairs of electrodes.

Listing 8 presents how to extract bioelectrical signals from electrode F4 for the participant with identity 1, observed by the EEG channel. The query was an instance of the generic CQ3. Referring to that query, the signal was not identified by signal_id, but it was searched by the specific pattern (the first two elements of the MATCH clause). Correspondingly, on the same principle, signal data from ECG and GSR channels were retrieved and merged to the one training data frame.

**Listing 8.** Extraction of F4 electrode signal for the dataset creation.
MATCH (p:Participant {user_id:1})-[:TAKES_PART_IN]-(r:Recording)-[:PART_OF]-(e:Experiment {name:’CS1’}),

(r)-[:HAS_RECORDING_TYPE]-(c:Channel {type:’EEG’})-[:HAS_MEASURE]-(m:ElectrodeMeasure {name:’F4’}),

(m)-[:HAS_SIGNAL]->(s1_root:SignalValue)-[:NEXT*0..]->(s1:SignalValue)<-[:IN_SEC]-(startTimestamp:Timestamp)

OPTIONAL MATCH (s1:SignalValue)<-[:END_IN_SEC]-(endTimestamp:Timestamp)

RETURN startTimestamp.value, endTimestamp.value, s1.value


In this short study, a classification task was conducted, thus every value of valence and arousal were converted to the appropriate classes. For example, if both valence and arousal were greater than zero, these values were converted to the High Valence—High Arousal (HVHA) class. This way, we had also Low Valence—Low Arousal (LVLA), High Valence—Low Arousal (HVLA), and Low Valence—High Arousal (LVHA) classes. Target values were based on the external annotations data, originating from the Amigos dataset.

In the next step, the dataset was split into train and test sets with the stratified shuffle split mechanism. This ensured the same distribution of classes both in training and testing sets. The training set size was set to 80% of the entire dataset. All categorical classes were one-hot encoded for the purposes of the softmax classifier. What is more, bioelectrical signals were normalized with the Min-Max scaler, to ensure that signals of one type did not have much of an effect on the neural network, just because they had numerically greater values.

The network input takes a vector with 181 values representing the calculated properties from bioelectrical signals. Its architecture would be considered rather simple, but in this case, fewer layers provided better generalization. The network consisted of two 64 dense layers with ReLu activation function, a dropout layer of 20%, two 128 dense layers again with ReLu as the activation function, and one more dropout. Finally, the network had the last layer with softmax activation function, which was dense 4, since we classified signals into four classes. The selected optimizer was Adam, and the loss function is the categorical cross-entropy. Selected metrics were accuracy and F1-score. Training took 50 epochs.

The results on the test set with a precision of 0.61 and an F1-score of 0.59 prove that the GRISERA framework allowed for storing data from external experiments and then retrieving it to train machine learning models. However, it is important to emphasize the metrics should not be compared to the results of other studies, as the purpose of this validation was only to demonstrate the feasibility of using the proposed framework for data storage and retrieval.

### 8.4. AMIGOS, DEAP and ASCERTAIN Datasets for Statistical Analysis

The aim of the second validation scenario was to verify whether it was possible to integrate data originating from different experiments, i.e., AMIGOS, ASCERTAIN and DEAP. The goal of the integration was to prepare data for statistical analysis allowing to compare various measures with respect to emotional states. Thus, it was assumed that the epoch length for all represented biosignals and emotional states was the same and in this scenario equal to exactly 10 s (choosing the best epoch length for statistical analysis was out of the scope of this scenario). For the datasets the following data were integrated:

- emotional states in Ekman model—for AMIGOS database emotional states were recognized by Face Reader, with a constant sampling rate of 1 reading per second. The signal was then divided into epochs and averaged. The same technique was used for ASCERTAIN. Here, the emotional state data was provided at different frequencies for different participants—most of the recordings had a frequency of 20 Hz, while a select few had a frequency of 25 Hz—corresponding to the different framerates of the video recordings. According to GRISERA principles, the values were preprocessed and again averaged over same-length epochs in order to solve the problem of the difference in frequencies. The values for emotional states are represented in range 0 to 1 inclusive. For DEAP database the emotional states in the Ekman model were not provided.
- GSR signals and ECG signals—no issues were found while storing them.
- EEG signals—the AMIGOS and DEAP datasets used a different number of EEG electrodes for measurements. However, both used the same naming and positioning system which allowed for easy comparison based on overlapping nomenclature. ASCERTAIN used a commercial-grade single-electrode EEG biosensor.

The data from the various experiments were extracted for analysis from the graph database using Cypher queries. The analysis was performed using Tableau with the use of an existing web data connector. The Cypher queries used to extract the data in a tabular form—fit for further analysis—are shown in Listing 9. This is a generic query for a signal of a given channel type (<channelType> parameter) and *n* different signal values of interest. Said values are denoted as parameters “signal_value_1”, “signal_value_2”, ..., “signal_value_n”. The resulting tabular data would contain not only the signal values but also related metadata and timestamps, resulting in n+7 columns. The query, with analogy to generic query CQ3, retrieved signal values, however not for one signal only (identified with signal_id), but for the whole set of signals matching the specified pattern.

**Listing 9.** Generic data extracion Cypher query for Tableau.
MATCH
  (r:Recording)-->(c:Channel {type:<channelType>})-->(e:Electrode Measure)  -->(s_root:SignalValue)-[:NEXT*0..]->(s:SignalValue)  <-[:START_IN_SEC]-(t_start:Timestamp),  (s)<-[:END_IN_SEC]-(t_end:Timestamp),   (x:Experiment)--(r)--(p:Participant)
RETURN
  x.id AS experiment,  r.source AS source_database,  p.id AS participant,  c.type AS channel_type,  e.name AS measure_name,   s.signal_value_1 AS signal_value_1,   s.signal_value_2 AS signal_value_2,  s.signal_value_3 AS signal_value_3,   ...,  s.signal_value_n AS signal_value_n,   t_start.value AS epoch_start,  t_end.value AS epoch_end

The scenario proved that it was possible to represent data from various datasets in GRISERA. It also proved that some of the data were well integrated and easy to retrieve for statistical analysis, which was done with Tableau tool. An example of such analysis is the average number of GSR peaks per second versus emotional states for AMIGOS and ASCERTAIN.

The analogical analysis can be performed for ECG signal or EEG signal. However, the latter one can be easily done only for the same electrode positioning system (in our case for AMIGOS and DEAP), as the values relate to the specified electrodes.

## 9. Discussion

In this paper, it has been proved that the use of graph data representation makes it possible to retrieve data from different experiments in a unified way (RQ1). It was achieved by:

- providing GRISERA—the unified graph representation for experiment originated data,
- providing the generic queries allowing to retrieve data from various signals,
- applying the GRISERA framework in the two case studies, where the specific instances of generic competency questions were used.

At this point it is important to emphasize the fact, that the GRISERA framework is an integration framework—it means that using GRISERA, experiment originated data can be uploaded, unified and retrieved (analogically, as in other integration solutions like data warehouses). The presented research proves the validity of such integration and opens possibilities to expand GRISERA and use it for various applications. The flexibility and expandability of GRISERA allows to store signals in the source form, as well as in the processed one, depending on the applicational needs. The explicit distinction between timestamp signal and epoch signal sets the semantics of the measure. The diverse contextual information can be modelled in the form of properties assigned to the specified nodes. This feature can be used for various application of GRISERA like storing the location of video or sound recordings or experiment versions. The set of information stored in GRISERA mostly depends on its application.

It has been also proved that the graph representation of the relationship between biosignals, identified emotional states, and contextual information allow for the extraction of useful data for Affective Computing research (RQ2). This aim was achieved by:

- designing the competency questions allowing to search for the signals described with the specified contextual information and retrieving their values,
- application of the GRISERA framework in the real AC applications.

Although the ability of data extraction from the graph is obvious, the range of extraction depends mainly on the built relationships. Thus, the contextual information, biosignals and emotional states are related on several levels. Firstly, signal values are related to each other by the common timeline. It allows to extract values depending on other ones (originated from other biosignals or estimated emotional states). Biosignals and emotional states are related to each other by channels, recordings and experiments introduced within contextual information.

Both research questions regard the problem of data extraction. It was shown that using Cypher queries it is possible to retrieve useful data in a unified way. Still, it is worth mentioning, that the Cypher language is well integrated with various programming languages as well as libraries that allow for advanced data storage and processing. There are additional drivers for many popular programming languages (including Java, Python, R, .NET, Haskell etc.), that mimic existing database driver semantics and approaches. What is more, it is possible to connect with the Neo4j/Cypher interface using HTTP-API. In this way, it is feasible to retrieve data from a graph database and save them to the data frame, e.g., using the Python language and the Pandas or Spark libraries.

The GRISERA framework is a novel approach in Affective Computing by:

- introducing the unified representation for contextual information, signal values and emotional states (it combines in one representation experimental and computational data),
- deployment of graph representation for all these types of data (not only specific types of signals),
- providing the unified way of retrieving various types of data for AC research.

The framework, as every solution, is intended to address the specific need. As a consequence, some features of the GRISERA can be seen as limitations. The basis of the design of the framework is data integration and reusability. These assumptions led us to define the framework for representing various experiment originated data. Therefore, it must be emphasized, that the solution is not intended for the specific applicational use, but for providing the ability to retrieve the data in a unified way for various applications as well as for producing new datasets. The framework is intentionally not optimized neither for statistical queries nor any other applicational use (in contrast to e.g., data warehouses, which integrate data for the specific analytical application). Using this framework demands retrieving data and consequently processing them in dedicated solutions for specific applications. The consequence of data reusability is the usage of the specified representation, which, as such, demands data description according to some rules and often introduces additional workload. Thus, the GRISERA usage may be inconvenient when data are prepared just for the single specific research and are not intended to be further (re)used or shared.

The other limitations arise directly from the placing GRISERA in the context of Research Data Universe [5]. GRISERA is just a single building block in the whole structure that must exist to increase the maturity of resources in community data collections. Within this idea, not only the GRISERA must evolve to include new features but also the other elements of the universe must be developed. These aspects, presented as future work, are contained in Section 10.

## 10. Conclusions

The framework proposed in this paper addresses the nagging problem of data integration and reusability in Affective Computing. To discuss the future development of the GRISERA framework it is important to perceive this framework as an element of wider initiative leading to build the mature solutions for data reusability in AC community. Thanos [5] identified various aspects of data reusability, which influence the further development of GRISERA.

1. Derivative use, allowing building new datasets on preexisting ones, sets the path of GRISERA development related to building automatic, configurable methods of signal transformations e.g., changing signal type from timestamp to epoch or changing epoch length,
2. Reusable lexicons providing the set of linguistic terms, points at the development of reusable lexicons in AC, which should be further incorporated in contextual data of GRISERA framework to support the data integration and new dataset creation,
3. Reusable ontologies, defining the relationships among the object, should be the base of semantics validation for the GRSIERA graph. Moreover, the contextual data should be compilant with standard ontologies for contextual data allowing to easily search for needed experiments, signals and other data.

We are conscious that the GRISERA framework should be also encapsulated with the technological stack, allowing to use them by the researchers, without deep knowledge about the internal and technical aspects of the presented solution.

## Figures and Tables

**Table 1:** Memory efficiency for Neo4J.

Contents	Record Size
Nodes	15 B
Relationships	34 B
Properties for nodes and relationships	41 B
Values of string properties	128 B
Values of array properties	128 B

## Data Availability

Not applicable.

## Abbreviations

   The following abbreviations are used in this manuscript:

AC	Affective Computing
ECG	Electrocardiography
EEG	Electroencephalography
ERD	Entity Relationship Diagram
ETL	Extract-Transform-Load
IC	Integrity constraints
GSP	Graph Signal Processing
GSR	Galvanic Skin Response
PSD	Power Spectral Density

## Appendix A. Graph Elements Semantic

**Table A1:** *VerLabs* elements.

Element	Element Description
Channel	observation of the participation of the specified participant by the specified channel
Electrode Measure	recorded measure obtained from the specified channel, which can influence on the other measure
Epoch measure	recorded measure obtained from the specified channel, which values are settled for the whole epoch
Experiment	conducted experiment
Measure	Electrode Measure or Epoch Measure or Timestamp Measure
Participant	participant of the experiment
Recording	participation of the specified participant within the specifed experiment
Signal Value	value of the specified measure
Timestamp	distance from the begining of the recording
Timestamp Measure	recorded measure obtained from the specified channel, which values are detected in the specified moment of time

**Table A2:** *EdgeLabs* elements.

Element	Element Description
END_IN_SEC	indication on the timestamp ending the epoch
HAS_MEASURE	assignment of the measure to the channel
HAS_RECORDING_TYPE	assignment of the channel to the recording
HAS_SIGNAL	indication on the first value in the series for each type of measure and timeline
INFLUENCE_ON	influence of one value on the other one
IN_SEC	indication on the timestamp, in which the value is detected for timestamp series
NEXT	succession of timestamps or signal values
PART_OF	assignments recording as a part of the experiment
START_IN_SEC	indication on the timestamp starting the epoch
TAKES	indication on the timeline for the experiment
TAKES_PART_IN	taking part in the recoring by the participant

**Table A3:** The settled subset of *Prop* elements.

hltextbfProperty	**Property Description**	**Possible Values**
age	age of the participant	Integer
alpha	the power of EEG signal within 8-12 Hz	Float: <100
beta	the power of the EEG signal within 12-30 Hz	Float: <100
datatype	data type for timestamp and epoch measure	String
delta	the power of EEG signal within 0,5-4 Hz	Float: <100
gamma	the power of EEG signal within 30-100 Hz	Float: <100
gender	gender of the participant	String: male, female or unknown
GSR_avg	average value of GSR for the specified epoch	Float
GSR_max	maximum value of GSR for the specified epoch	Float
GSR_min	minimum value of GSR for the specified epoch measure	Float
HRV	average heart rate variability for the specified epoch expressed in ms	Integer
ibi	average interbeat interval for the specified epoch expressed in ms	Integer
mi	mutual information	Float
name	name of the experiment and each type of measure	String
no_of_peaks	no of GSR peaks within the specified epoch	Integer
range	range of values for timestamp and epoch measure	String
recording_standard	recording standard for the specified channel	String
signal_id	signal identifier	Integer
theta	the power of EEG signal within 4-8 Hz	Float: <100
type	type of channel	String, e.g., audio, BVP, chest size, depth video, ECG, EEG, EDS, EMG, GSR, RGB video, temperature
user_id	identifier of the participant	Integer
value	value of the timestamp and signal value	simple value for epoch measure and timestamp measure, list of original values for electrode measure

## Appendix B. Integrity Constraints

The full list of mandatory properties ICs is defined in Table A4. The label specifies the set of nodes or properties, for which the constraint is valid and the property specifies for which property, the value must be specified with a non-null value.

**Table A4:** Mandatory properties ICs.

Label	Property
Channel	type
Epoch Measure	datatype
Epoch Measure	range
Experiment	name
INFLUENCE_ON	mi
Measure	name
Measure	signal_id
Participant	user_id
Signal Value	value
Timestamp	value
Timestamp Measure	datatype
Timestamp Measure	range

Additionally to the constraints possible to define in the Cypher query, the property value limitations ICs must be kept when creating the GRISERA graph. The possible values of properties are defined in Table A3.

Two more types of ICs (i.e., cardinality and endpoint constraints) are defined in Table A5. The used notation is similar to the one used in ERD diagrams (1 denotes that there is exactly one edge, 0..1 that there is at most one edge and * zero or more edges). Each row of the Table A5 represents the required cardinality between subject and object (both understood as the particular nodes with the specified labels) as connected by the edges with the specified edge label. Object cardinality refers to the possible number of objects the subject can be connected to, while subject cardinality refers to the possible number of subjects being connected to the specified object. For example, the first row of the Table A5 states that one node with the Participant label can be connected to numerous nodes with the Recording label with edges with the TAKES_PART_IN label while in the same time each Recording node can be connected to the single Participant node only.

**Table A5:** Cardinality and endpoint ICs.

Subject	Subject Cardinality	Egde Label	Object Cardinality	Object
Channel	1	HAS_MEASURE	*	Timestamp Measure
Channel	1	HAS_MEASURE	*	Epoch Measure
Channel	1	HAS_MEASURE	*	Electrode Measure
Electrode Measure	0..1	HAS_SIGNAL	1	Signal Value
Epoch Measure	0..1	HAS_SIGNAL	1	Signal Value
Experiment	*	TAKES	1	Timestamp
Participant	1	TAKES_PART_IN	*	Recording
Recording	*	PART_OF	1	Experiment
Recording	1	HAS_RECORDING_TYPE	*	Channel
Signal Value	0..1	NEXT	0..1	Signal Value
Signal Value	*	INFLUENCE_ON	*	Signal Value
Timestamp Measure	0..1	HAS_SIGNAL	1	Signal Value
Timestamp	0..1	NEXT	0..1	Timestamp
Timestamp	1	IN_SEC	*	Signal Value
Timestamp	1	START_IN_SEC	*	Signal Value
Timestamp	1	END_IN_SEC	*	Signal Value

## References

[1] Picard R.W. (2000). Affective Computing.

[2] Poria S., Cambria E., Bajpai R., Hussain A. (2017). A review of affective computing: From unimodal analysis to multimodal fusion. Inf. Fusion.

[3] Shuman D.I., Narang S.K., Frossard P., Ortega A., Vandergheynst P. (2013). The emerging field of signal processing on graphs: Extending high-dimensional data analysis to networks and other irregular domains. IEEE Signal Process. Mag..

[4] Sandryhaila A., Moura J.M. (2014). Big Data Analysis with Signal Processing on Graphs: Representation and processing of massive data sets with irregular structure. IEEE Signal Process. Mag..

[5] Thanos C. (2017). Research Data Reusability: Conceptual Foundations, Barriers and Enabling Technologies. Publications.

[6] National Research Council (1997). Bits of Power: Issues in Global Access to Scientific Data.

[7] National Research Council (2000). A Question of Balance: Private Rights and the Public Interest in Scientific and Technical Databases.

[8] Simberloff D., Barish B., Droegemeier K., Etter D., Fedoroff N., Ford K., Lanzerotti L., Leshner A., Lubchenco J., Rossmann M. (2005). Long-Lived Digital Data Collections: Enabling Research and Education in the 21st Century.

[9] Lim K.T., Maier S.D., Ratzesberger O., Zdonik S. Requirements for science data bases and SciDB. Proceedings of the Conference on Innovative Data Systems Research.

[10] Wickett K.M., Sacchi S., Dubin D., Renear A.H. (2012). Identifying content and levels of representation in scientific data. Proc. Am. Soc. Inf. Sci. Technol..

[11] Pokorny J. (2016). Conceptual and Database Modelling of Graph Databases.

[12] Francis N., Taylor A., Green A., Guagliardo P., Libkin L., Lindaaker T., Marsault V., Plantikow S., Rydberg M., Selmer P. (2018). Cypher: An Evolving Query Language for Property Graphs.

[13] Correa J.A.M., Abadi M.K., Sebe N., Patras I. (2018). AMIGOS: A Dataset for Affect, Personality and Mood Research on Individuals and Groups. IEEE Trans. Affect. Comput..

[14] Subramanian R., Wache J., Abadi M.K., Vieriu R.L., Winkler S., Sebe N. (2018). ASCERTAIN: Emotion and Personality Recognition Using Commercial Sensors. IEEE Trans. Affect. Comput..

[15] Koelstra S., Muhl C., Soleymani M., Lee J.S., Yazdani A., Ebrahimi T., Pun T., Nijholt A., Patras I. (2011). Deap: A database for emotion analysis; using physiological signals. IEEE Trans. Affect. Comput..

[16] Soleymani M., Lichtenauer J., Pun T., Pantic M. (2011). A multimodal database for affect recognition and implicit tagging. IEEE Trans. Affect. Comput..

[17] Katsigiannis S., Ramzan N. (2017). DREAMER: A database for emotion recognition through EEG and ECG signals from wireless low-cost off-the-shelf devices. IEEE J. Biomed. Health Inform..

[18] Becker H., Fleureau J., Guillotel P., Wendling F., Merlet I., Albera L. (2017). Emotion recognition based on high-resolution EEG recordings and reconstructed brain sources. IEEE Trans. Affect. Comput..

[19] Song T., Zheng W., Lu C., Zong Y., Zhang X., Cui Z. (2019). MPED: A multi-modal physiological emotion database for discrete emotion recognition. IEEE Access.

[20] Seal A., Reddy P.P.N., Chaithanya P., Meghana A., Jahnavi K., Krejcar O., Hudak R. (2020). An EEG Database and Its Initial Benchmark Emotion Classification Performance. Comput. Math. Methods Med..

[21] Shu L., Xie J., Yang M., Li Z., Li Z., Liao D., Xu X., Yang X. (2018). A review of emotion recognition using physiological signals. Sensors.

[22] Siddharth S., Jung T.P., Sejnowski T.J. (2019). Utilizing deep learning towards multi-modal bio-sensing and vision-based affective computing. IEEE Trans. Affect. Comput..

[23] Goodwin A.J., Eytan D., Greer R.W., Mazwi M., Thommandram A., Goodfellow S.D., Assadi A., Jegatheeswaran A., Laussen P.C. (2020). A practical approach to storage and retrieval of high-frequency physiological signals. Physiol. Meas..

[24] Song C., Qin X.H., Zhou Q., Wang Z.Y., Liu W.H., Li J., Huang L., Chen Y., Tang G., Zhao D.J. (2018). PlantES: A plant electrophysiological multi-source data online analysis and sharing platform. Appl. Sci..

[25] Chen Y., Wang Z.y., Yuan G., Huang L. (2017). An overview of online based platforms for sharing and analyzing electrophysiology data from big data perspective. Wiley Interdiscip. Rev. Data Min. Knowl. Discov..

[26] Thürk F., Kampusch S., Kaniusas E. (2015). Management framework for biosignals in biomedical studies: From study design to data statistics. IEEE Trans. Instrum. Meas..

[27] Kokkinaki A., Chouvarda I., Maglaveras N. (2012). Searching biosignal databases by content and context: Research Oriented Integration System for ECG Signals (ROISES). Comput. Methods Programs Biomed..

[28] Carreiras C., Silva H., Lourenço A., Fred A. Storagebit-a metadata-aware, extensible, semantic and hierarchical database for biosignals. Proceedings of the International Conference on Health Informatics.

[29] Abdulla S., Diykh M., Laft R.L., Saleh K., Deo R.C. (2019). Sleep EEG signal analysis based on correlation graph similarity coupled with an ensemble extreme machine learning algorithm. Expert Syst. Appl..

[30] Huang W., Bolton T.A., Medaglia J.D., Bassett D.S., Ribeiro A., Van De Ville D. (2018). A graph signal processing perspective on functional brain imaging. Proc. IEEE.

[31] Richiardi J., Achard S., Bunke H., Van De Ville D. (2013). Machine learning with brain graphs: Predictive modeling approaches for functional imaging in systems neuroscience. IEEE Signal Process. Mag..

[32] Lotte F., Bougrain L., Cichocki A., Clerc M., Congedo M., Rakotomamonjy A., Yger F. (2018). A review of classification algorithms for EEG-based brain–computer interfaces: A 10 year update. J. Neural Eng..

[33] Angles R., Arenas M., Barceló P., Hogan A., Reutter J.L., Vrgoc D. (2016). Foundations of Modern Graph Query Languages. arXiv.

[34] Angles R. The Property Graph Database Model. Proceedings of the 12th Alberto Mendelzon International Workshop on Foundations of Data Management.

[35] Ramesh D., Sinha A., Singh S. Data modelling for discrete time series data using Cassandra and MongoDB. Proceedings of the 2016 3rd International Conference on Recent Advances in Information Technology (RAIT).

[36] Damyanov D. (2018). Building a Model for Event Data as a Graph. https://snowplowanalytics.com/blog/2018/03/26/building-a-model-for-atomic-event-data-as-a-graph/.

[37] Brodny G., Kolakowska A., Landowska A., Szwoch M., Szwoch W., Wrobel M.R. Comparison of selected off-the-shelf solutions for emotion recognition based on facial expressions. Proceedings of the 2016 9th IEEE International Conference on Human System Interactions (HSI).

[38] Russell J.A. (1980). A circumplex model of affect. J. Personal. Soc. Psychol..

[39] Fornito A., Zalesky A., Breakspear M. (2013). Graph analysis of the human connectome: Promise, progress, and pitfalls. Neuroimage.

[40] Dictionary M.W. (2021). Merriam-Webster. www.merriam-webster.com.

[41] Sharma M., Kacker S., Sharma M. (2016). A brief introduction and review on galvanic skin response. Int. J. Med. Res. Prof..

[42] Gamboa H. (2008). Multi-Modal Behavioral Biometrics Based on HCI and Electrophysiology. Ph.D. Thesis.

[43] Lourenço A., Silva H., Leite P., Lourenço R., Fred A. Real Time Electrocardiogram Segmentation for Finger based ECG Biometrics. Proceedings of the International Conference on Bio-inspired Systems and Signal Processing—BIOSIGNALS.

[44] Thomson D. (1982). Spectrum estimation and harmonic analysis. Proc. IEEE.

[45] Slepian D. (1978). Prolate Spheroidal Wave Functions, Fourier Analysis, and Uncertainty-V: The Discrete Case. Bell Syst. Tech. J..

[46] Cox D.D. (1996). Spectral Analysis for Physical Applications: Multitaper and Conventional Univariate Techniques. Technometrics.

[47] Webber J. (2019). Neo4j for Very Large Scale Systems. https://www.youtube.com/watch?v=BfPDZf2wmqg.

[48] Santamaria-Granados L., Munoz-Organero M., Ramirez-Gonzalez G., Abdulhay E., Arunkumar N. (2018). Using deep convolutional neural network for emotion detection on a physiological signals dataset (AMIGOS). IEEE Access.

